# Dr. Henry A. Record

**Published:** 1917-08

**Authors:** 


**Dr. Henry A. Record,** Hahnemann, Philadelphia, 1865, died at his home in Forestville, April 22, of heart disease.

